# Systemic relationships between ecological-dynamic approach and active reflection: psycho-neuro-motor, cognitive, and physiological interactions in rugby pedagogy

**DOI:** 10.3389/fspor.2026.1804050

**Published:** 2026-05-14

**Authors:** Marta Rigon, Raffaele Scurati, Giampiero Merati, Damiano Formenti, Athos Trecroci, Pietro Luigi Invernizzi, Gabriele Signorini

**Affiliations:** 1 Department of Biomedical Sciences for Health, Università Degli Studi di Milano, Milan, Italy; 2 Department of Biotechnology and Life Sciences, University of Insubria, Varese, Italy; 3 IRCCS Fondazione don Carlo Gnocchi, Milan, Italy

**Keywords:** conscious state, constructivist approach, contemporary learning, counterfactual experiment, embodied awareness, systems thinking

## Abstract

**Introduction:**

The rugby pedagogy approach in the concept of System Thinking can be seen as multifaceted elements that interact together interconnected by a red thread of neuromotor-psycho-physical and cognitive aspects. The ecological dynamic approach can build the athlete/player's behavior and learning by inducing adaptive and self-organized systems resulting from lived experiences. The study aims to evaluate if an ecological and reflective approach applied to rugby pedagogy can enhance the coordination, the metabolic condition, can decrease the general fatigue and can help in managing the executive functions. Furthermore, the interrelationship between the coordination and metabolic condition, the coordination and the executive functions, the coordination and general fatigue were investigated.

**Methods:**

Forty boys U14 rugby players aged 12 to 14 years were enrolled for the study. Twenty boys were enrolled in ecological dynamic training (EXP), and twenty boys followed a traditional training (CON). The players' neuro-motor-psycho-physical and cognitive competences were investigated using KTK test, Yo-Yo IR1 test, Attention and Concentration test, Flanker task and their workload was measured with NASA-TLX.

**Results:**

The EXP group increased neuromotor, cognitive and perceptive capacities compared to CON group. No differences between groups were found in physiological aspects.

**Discussions:**

Through a counterfactual thought experiment, it was highlighted how the integration between the Ecological-Dynamic approach and the Relational Constructivist approach is essential for their real effectiveness in rugby pedagogy. Rugby pedagogy is a structural prerequisite for addressing contemporary ethical, health, and environmental challenges to educate for Embodied Awareness through Contemporary Learning.

## 1 Introduction

System thinking is a multifaceted and multilevel model, in which every element is interconnected with another, and every small change or variation in its part can alter the entire system (1, 2). These deep interrelationships can be seen as a “red thread” that connects an individual's experiences, as reported in ancient metaphors, binding “the world” together. The system thinking structure can be represented as a “matryoshka effect”, in which every level of the system is contained in another, and they are all dependent on the others, stabilized by a feedback circuit (3).

To the best of our knowledge, the ecological approach best aligns with the concept of system thinking, as it refers to the interactions between the organism, tasks and environment, including their constraints and affordances. The underlying relationships linking these elements can alter and modify the developing individuals's behavior and learning processes (4–8). The athlete/player's behavior and learning are induced by adaptive and self-organized systems resulting from the experiences done (9–11). This way, Gibson's theories view individuals as complex systems continuously interacting with environment (12). The dynamic ecological theory, rooted in ecological psychology and dynamic systems theory, emphasizes the mutual relationship between perception, action, and the individual-environment interaction (13, 14). This theory explains the complexity, variability, and nonlinearity inherent in learning processes, emphasizing how individuals adapt through continuous interaction with both external and internal stimuli (12). Learning is seen as a nonlinear process where the individual perceives and acts based on environmental opportunities. As individuals explore their environment, they develop new actions that are more effective and adaptable to the context (15). The coach's role is to facilitate this learning process by modifying constraints between the environment, activity, and individual (9). Rather than teaching fixed techniques, this pedagogy helps players discover methods that align with their unique abilities (16). Learning emerges from adapting actions to environmental constraints and goals, not from instructions or random exploration (12).This pedagogical approach, that is aligned on the concept of “Rugby Man,” (an educational vision of sport promoted by World Rugby) (17) promotes autonomy and competence, fostering a holistic view of the human being in relation to their environment, according to with the principles of Sport Pedagogy (18–20).

In particular, in team sport, several teaching methods focused on game tactics and strategies (Game-Centered Approach) follow this pedagogical vision based on models rooted in psychological and social constructivist theories of learning (21). These approaches emphasize active learning, where individuals actively seek and test information while considering their environment. Learning is seen as a mental construction process, integrating new knowledge with prior experience, and is situated within a socio-cultural context that shapes the learning process (22). Additionally, constructivist theory suggests that part of learning may be implicit and unconscious (23). The body is viewed not just as a vessel for learning, but as an integral component of the learning process, primarily through sensory and neuro-muscular systems. These ideas align with the TGfU approach, which prioritizes cognitive, tactical, and strategic reflection over technical skills, focusing on game engagement (22, 24). This way, the instructors should continuously reinforce technical and tactical understanding through problem solving questions and dialogue (active reflection) or guiding players to adapt directly to the environment (perception-action), in the absence of reflective processes, with the correct dosage (dose-response) of methodological approaches in relation to the needs (12, 25–27).

From a pedagogical perspective, the concept of rugby pedagogy suggests training models that combine highly variable practice environments with moments of cognitive reflection, thereby promoting the development of adaptive and transferable skills (12, 28, 29). The ecological dynamics approach is grounded in the manipulation of constraints—related to the organism, the task, and the environment—which can modify both task difficulty and complexity (30, 31). As in a complex system (systems thinking), changes in constraints, and consequently in complexity and difficulty, are reflected in the other elements of the ecological dynamics framework (redundancy, metastability, degeneracy, affordances, attractors, and attunement), altering their dosage-response and relative weighting (32).

In this nonlinear and integrated reflective pedagogy in rugby:

- constraints refer to changes in the environment, task and organism that induce stimulation of new and creative motor responses (33). Environmental constraints may include reducing pitch size, using soft or lighter balls, or organizing games in small-sided formats (e.g., 3vs3 or 4vs4) to increase ball involvement and decision-making opportunities;
- redundancy represents a concept of motor abundance concerning the use of different modalities and equivalences through which it is possible to carry out the same motor skill (34). Practice designs that value outcome effectiveness rather than technical uniformity encourage children to explore diverse motor solutions, supporting creativity and confidence;
- metastability represents in motor execution a balance between stability and instability, and is a condition of passage in the final motor control of a skill (14). Metastability in youth rugby emerges during transitional moments in play, such as when a ball carrier approaches a defender and must decide whether to pass, run, or slow down. Training games that allow time and space for decision-making—without immediate prescriptive instruction—help young players remain in this balance between stability and instability;
- degeneracy represents a lack of specialization of the human organism, characterized by a high number of motor possibilities guaranteed by the high number of degrees of freedom (35). Players of different sizes or physical capacities can successfully perform the same task (e.g., tackling, ball-carrying, or defending space) using different coordination strategies. Exposure to varied roles and positions during training promotes functional variability and reduces the risk of early specialization;
- affordances represent the opportunities of interpretation of a motor task that a situation determines in a subject in relation to his abilities and characteristics (36). An open space may afford running, while a nearby teammate affords passing or support play. Importantly, affordances are relative to the child's current abilities; therefore, scaling the game (pitch size, number of players, rules) is essential to ensure that relevant action possibilities are perceivable and actionable for young athletes;
- attractors represent a preferential state of stability that the organism tends to reach and maintain as a personal interpretation of an ability (14, 33). While these tendencies provide initial stability, training environments should gently perturb rigid attractors by introducing variability and guided questioning, encouraging children to expand their behavioral repertoire without inducing cognitive overload;
- multistability is the ability to adapt unexpected changes in a situation, in environmental variables or changes in one's organism (for example, fatigue). It assumes high self-control and stability of performance under different and not favorable conditions (37). For example, a child may learn to slow down play when tired or choose safer passing options under pressure. Progressive exposure to variability—while maintaining psychological safety—supports emotional regulation, self-control, and consistent performance across contexts;
- attunement represents the organism's ability through experience, to tune the perceptual process, making it progressively more sensitive to relevant information such as opportunities for action (38). Through repeated exposure to representative play and reflective dialogue (e.g., simple questions about what they noticed), children become more sensitive to meaningful cues that guide effective action, strengthening perception–action coupling.

**Figure 1:**
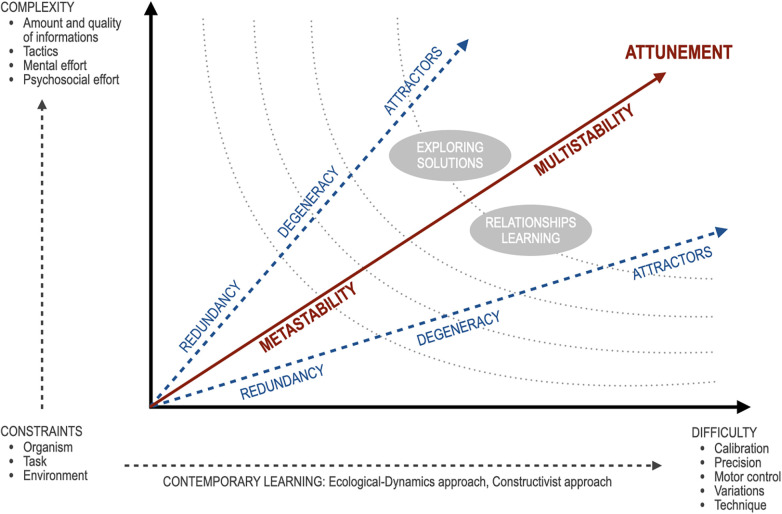
Representation of relationships between the elements that characterize the ecological dynamic and constructivist approach. The nature of the dynamic ecological approach is based on changes in constraints (organism, task, and environment), which can alter difficulty and complexity of the task. Dotted curved lines represent different levels of psychomotor load (dose-response).

The proportions and interactions established among these elements can influence learning outcomes, orienting the player either toward the exploration of multiple solutions or toward learning the relationships among existing elements to be able to interpret them considering one's psychomotor conditions of progress or regression in the different periods of training. Figure 1 illustrates the relationships among the elements that characterize the ecological dynamics and constructivist approach as an integrated vision.

An appropriate balance of task complexity and difficulty, achieved by acting on the various elements that constitute an integrated vision of rugby pedagogy, enables the coach to tailor methodological teaching processes toward either technical or tactical components. This according to specific needs, thereby fostering a better understanding of the game's language and principles among players. Contemporary learning approaches, grounded in ecological and constructivist principles, also align with the objectives of article 33 of the Italian Constitution and other European and international organizations, which assigns sports a central role in supporting the educational success of children and ensuring the development of all dimensions of their personality (39–42).

On this basis, the developing individual can be considered a psychophysical unit, requiring a multidimensional analysis that integrates neuromotor, physiological, perceptual, and cognitive aspects (43). According to the studies by Myer et al. (44), a high level of physical conditioning facilitates more effective coordination. Literature consistently suggests that regular physical exercise plays a crucial role in enhancing cerebral perfusion, particularly in the prefrontal cortex, through mechanisms such as angiogenesis, which increases the number and extent of blood vessels, and by promoting the nervous system's plasticity. This enhanced plasticity allows for the reorganization and control of bodily actions to better adapt to various situations (45–48). Notably, the positive effects of physical conditioning in sports characterized by intermittent bouts of exercise and rapid changes in intensity are particularly well-documented during adolescence and pre-adolescence (49).

Furthermore, the American College of Sports Medicine recognizes coordination and perceptual abilities—classified as neuromotor skills—as essential components of health-related fitness. These abilities have significant beneficial effects on cardiovascular and metabolic function, disease prevention, and overall functional performance (50). The scientific literature indicates that coordination and perceptual skills develop in a non-linear manner, with significant influences from chronological and biological age, as well as environmental factors (51). Consequently, providing varied and diverse stimulation of these abilities throughout the lifespan is essential.

Children's and young people's development can be framed within a psychophysical model that emphasizes awareness, focusing on the individual as the core. This model integrates mental, physical, perceptual, and motor dimensions to support functional performance and overall well-being (52–55).

This way, substantial scientific evidence supports the effectiveness of game-centered approaches, grounded in ecological-dynamic and constructivist theoretical frameworks. These approaches demonstrate integrated and multifaceted effects, not only in the domains of motor coordination and fitness but also in cognitive and perceptual capacities, particularly those involving the interaction between the individual and their environment (56). Compared to traditional methods, game-centered approaches show superior outcomes in enhancing motor skills, fitness levels, and cognitive abilities such as decision-making, tactical awareness, and perceptual sensitivity—critical factors for individuals' interactions with their environment (57–60).

These findings emphasize the holistic nature of game-centered teaching strategies, which prioritize not only physical development but also the enhancement of cognitive and perceptual skills, essential for personal and social growth across various contexts. The integration of these approaches into educational and training settings can provide comprehensive benefits, fostering the development of both physical and cognitive competencies in dynamic, real-world contexts.

Through rugby pedagogy, psychophysical literacy can be developed by engaging both conscious and unconscious learning processes grounded in embodied and movement-based experience (16). Awareness arises from the integration of bodily perception, motor control, and attentional processes. This embodied self-awareness serves as a prerequisite for the development of broader and more global forms of awareness (61, 62), supporting adaptive interaction with others, the environment, and sport-specific contexts (63). Significant associations between physical activity and physical self-concept in youth have been reported, with self-awareness playing a crucial role in promoting physical activity, which has positive health implications (64). A synthesis of concepts and literature main findings is reported in the Supplementary Appendix 1.

**Figure 2:**
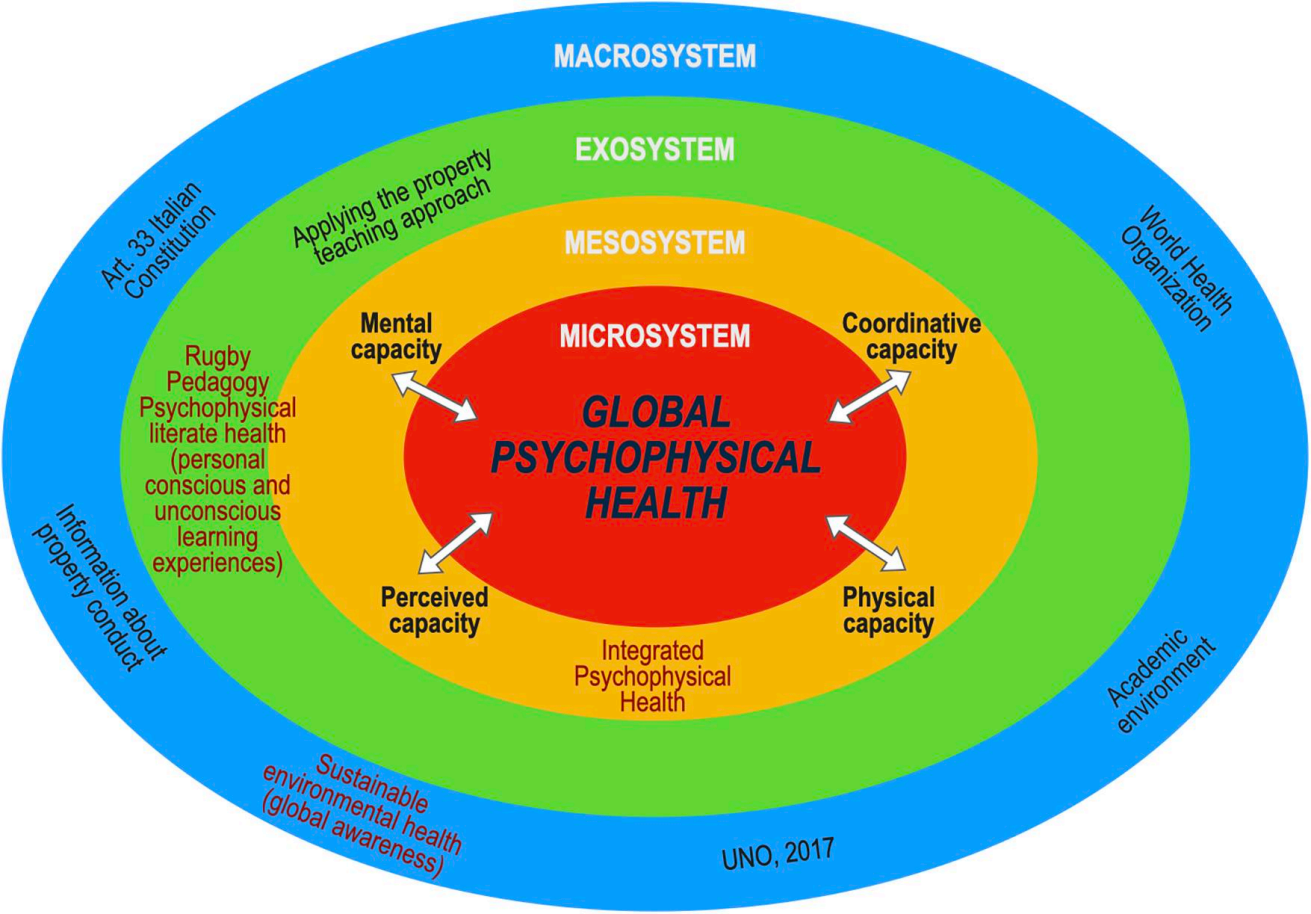
Representation of the concept of one health as integration of individual conscious and unconscious experiences with sustainability health based on global awareness.

In this context, educational processes align with national and international institutions and regulatory bodies that advocate for an ethical and holistic global vision, emphasizing personal responsibility, health, and sustainable development through sport and physical education (Figure 2) (65).

In the Italian context, rugby pedagogy has gained increasing importance in recent years, reflected in a significant rise in youth practitioners. Consequently, the number of rugby clubs has also increased, necessitating the establishment of a structured rugby pedagogy in not only major clubs but also smaller ones. Since the right to sports education is fundamental for all young people, this study aims to explore whether, within a typical rugby club: (a) an ecological and reflective approach to rugby pedagogy can enhance coordination (neuromotor aspects) and metabolic conditioning (physiological aspects), reduce the perception of various types of fatigue (perceptual aspects), and support the management of executive functions (cognitive aspects); and (b) the interrelationships between coordination (neuromotor aspects), metabolic conditioning (physiological aspects), executive functions (cognitive aspects), and perceptual aspects can be promoted by an ecological and reflective approach to rugby pedagogy.

## 2 Materials and methods

Forty boys aged 12–14 years from the U14 category of a rugby team in the hinterland of Milan, were enrolled for the study. Participants engaged exclusively in rugby training during their week, without involvement in any other sports. The involved rugby team had a solid structure, including all youth categories (Minirugby, U14, U16, U18). The club had a structured sports program and provided instructor education comparable to that of similar rugby teams in the area. Athletes participated in regional round tournaments, for which no annual ranking is assigned, in accordance with federal regulations, as well as in national and international tournaments. The inclusion criteria include having at least 4 years of rugby experience and the absence of injuries. The sample size satisfied the request of the minimum number of participants calculated with G*Power software (*N* = 36), Effect size f set at 0.25. Twenty players, attending the experimental group (EXP) underwent ecological and reflective approach applied to rugby pedagogy, while the other twenty players attending the control group (CON) underwent traditional training. The inclusion in EXP or CON group was determined by a random selection, performed using randomization software (https://www.random.org). Anthropometrics are reported in Table 1.

**Table 1:** Anthropometrics.

Group	Participants (*n*)	Age (yrs)	Weight (kg)	Height (cm)	BMI (kg/m^2^)
Experimental group (EXP)	20	13.2 ± 0.8	58.9 ± 8.2	164.8 ± 4.3	21.7 ± 2.7
Control group (CON)	20	13.1 ± 0.5	59.7 ± 7.3	164.7 ± 5.1	22.0 ± 1.9

No significant differences were observed in participants' anthropometric characteristics.

### 2.1 Procedures (timeline)

**Figure 3:**
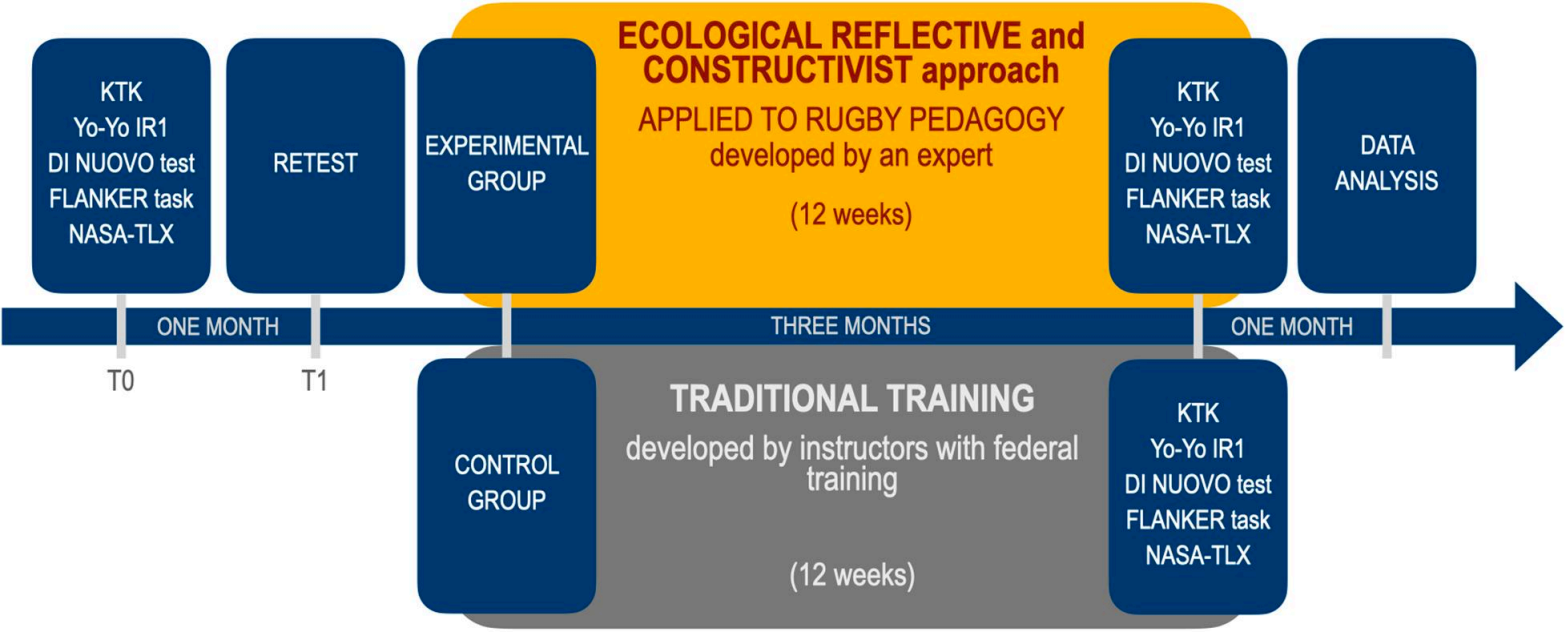
Timeline of the research.

The participants' coordination skills, aerobic conditioning and executive functions were tested before starting the experimentation (T0). At the end of each test, the players were invited to rate different types of perceived workload. After testing the reliability (T1), participants were divided into experimental (EXP) and control (CON) groups. The players of the experimental group were trained with the ecological and reflective approach applied to rugby pedagogy by an expert, whereas the players of the control group continued with traditional training program guided by a federal instructor. The intervention was conducted twice a week for 12 consecutive weeks, after which the players were retested as in T0. The timeline of the research is represented in Figure 3.

This study was approved by the ethical committee of the University of Milan (n. 14/24, 13.02.2024).

### 2.2 Training sessions

The training session included a general activation phase lasting about 20 min, in which players performed technical and physical exercises oriented to the basic game skills. After that the players were assigned to the different groups (EXP and CON). The experimental group was trained with the ecological and reflective approach applied to rugby pedagogy led by an expert for 45 min, the control group (CON) was trained with the traditional method for 45 min, led by a federal instructor. The sessions included moderate-to-vigorous-intensity exercise. The duration of the technical exercise (representing difficulty variable in Figure 1) ranged from 5 to 10 min, while small-sided and formal games (representing complexity variable in Figure 1) lasted 15 to 20 min. All the lessons were offered twice a week. The instructors' characteristics are reported in Table 2.

**Table 2:** Rugby instructors' comparison.

Instructor characteristics	Instructor using the ecological dynamics approach	Instructor using the traditional approach
Age (years)	37	30
Gender	F	M
Sport and teaching experience (years)	7	8
Federal Rugby Formation	Yes	Yes
Specific training on ecological and reflective approach	Yes	No
University formation (sport science graduation)	Yes	Yes
Doctorate on nonlinear pedagogy subject	Yes	No

The ecological and reflective training was characterized by rugby experiences aimed at the modification of the organism, environment, and tasks adding cognitive stimulation to increase the perceptive workload. Specifically, criteria of interventions are the following: the instructor limits her role to communicating the rules of the activity, promoting an external focus of attention (15). She encourages the exploration of multiple movement solutions and the implementation of problem-solving strategies. Technical explanations are provided only when required for safety-related aspects (as tackles didactics); in such cases, it is recommended to first prompt players' reflection to foster a more participatory approach. The instructor may also employ metaphors and imagery to stimulate diverse interpretations of game actions. Feedback should not be corrective in nature; rather, it should function as positive reinforcement of the action, preferably supported by an explanatory rationale (66). Within this framework, the instructor can use open-ended questioning to facilitate the autonomous identification of solutions, avoiding the provision of definitive answers. Attention is directed toward the most relevant stimuli through the proposal of “autonomy-supportive challenges” (e.g., “Where could you position yourselves to make your teammates' passing easier?”). Questions are intended to promote movement variability, in line with the principle of “repetition without repetition”. Task demonstration is not provided.

The traditional approach to rugby instruction is commonly associated with directive methodologies, in which the instructor represents the primary source of knowledge for the player. The criteria of interventions are the following: the instructor provides explanations of the technical and tactical aspects of the game, supported by explicit demonstrations (67). Learning is structured through a progression from simple to complex tasks (68, 69), with activities conducted in contexts isolated from the game. Systematic repetition of exercises is aimed at reducing execution errors. The instructional approach has been shown in the literature to produce positive effects on learning outcomes (70, 71). Feedback is corrective in nature and is initially delivered more frequently, while remaining focused on critical aspects and relevant issues (67). It may be directed toward either an external attentional focus (e.g., ball trajectory) or an internal attentional focus (e.g., increased knee flexion), in accordance with findings reported in the literature (72).

Examples of intervention contents of both approaches are reported in the Supplementary Appendix 2.

During our study, the players of both groups presented a positive social climate; they were collaborative with the expert and the instructor and followed the instructions given during all training sessions. The players shared a homogeneous and common background in rugby, with at least four or more years of prior experience.

### 2.3 Measures

To measure all the considered variables the following testing tools were used.

The *KTK Körperkoordinationstest für Kinder test* (73) was used to evaluate coordinative aspects. It consists of four trials (balance, single-leg jump, side jumps, lateral translocation), whose raw scores are transformed into motor quotients (MQ) based on gender and age, which are then summed to form a total MQ. The KTK has been validated for children from 5 to 15 years of age.

Given the performance pattern of rugby and the players' ages, the Yo-Yo IR1 (74) was used to evaluate physiological aspects. It is a shuttle test consisting of running 20 + 20 m and walking 10 m at incremental speed to exhaustion (inability to sustain the speed or abandonment of the test by the subject). This test has been validated also for individuals of 11–16 years of age (75).

Three tests from Di Nuovo's Attention and Concentration tool (76) assessed inhibition, working memory, and cognitive flexibility. Complex and straightforward inhibition are measured by asking the subject to press the computer's space bar when a star appears; working memory is measured based on rewriting sequences of numbers as they appear or in the reverse order; the cognitive flexibility measure comes from a task consisting of searching and eliminating from the screen the letters or pictures that appeared at the top. For each test, the number of errors and omissions was recorded. The test has been also previously used in participants aged 10–14 years (77).

The executive functions were measured by Flanker task (78), a inhibitory control and selective attention test where participants had to press the arrow left or right button on the computer keyboard to identify the direction of the arrows in the screen for congruent (left <<<<<; right >>>>>) or incongruent (left >><>>; right <<><<) conditions. One hundred items were shown (50 congruent, 50 incongruent conditions). For both congruent and incongruent conditions, the mean response time of the correct responses and response accuracy were considered for the analysis. The flanker effect variable (congruent reaction time—incongruent reaction time) was considered for the analysis.

To assess perceived workload the *NASA-TLX* was administered (79). It considers not only physical, but also the psychological aspect by investigating six domains: perceived mental, time, and physical demands and perceived performance, effort, and frustration. It is divided into Part A (weights), which considers the weight of each factor relative to the total workload of a specific task, and Part B (ratings), which uses scales to express the magnitude of each factor. The scales are represented graphically by 12 cm lines divided into 20 intervals by notches. Each notch is worth 5 points, ranging from 0 to 100, with 0 indicating the lowest value and 100 the highest (rounding up if a subject marks in the middle of an interval rather than on a notch). Once both data sets have been collected for each subject, the number of times an entry is circled is counted. The sum of all entries must equal 15. Each value must then be adjusted by multiplying each entry's number by its weight. The adjusted values must be summed and then divided by 15 to obtain the taskload for each subject (NASA Taskload Index). The questionnaire has been considered valid for participants aged 6 years or older (62, 80).

### 2.4 Statistical analysis

The Shapiro–Wilk test and the kurtosis/skewness analysis were performed to assess the normal distribution of the data. Data are shown as mean ± standard deviation (SD). Test-retest reliability was assessed using the intraclass correlation coefficient (ICC). The two-way (Time × Group) Analysis of Variance (ANOVA) with repeated measure on one factor (Time) was performed to assess the effects of the ecological dynamical approach on each variable. Magnitude of the effect was also quantified calculating *η*^2^ as Effect size. The time effect (pre vs. post), the group effect (experimental vs. control), and the interaction (time × group) were assessed for each variable. In case of significance, the Bonferroni *post-hoc* test was performed. Effect sizes (ESs, as Cohen's d values) were calculated to interpret the comparison between time, group and time × group. The analysis of the workload was assessed using ANOVA for repeated measures followed by the Bonferroni's *post-hoc* test in case of significance. Pearson's correlation coefficient was calculated to quantify the relationship between KTK, yo-yo IR1 and NASA-TLX demands.

The level of significance was set at *α* = 0.05 (*p* < 0.05).

### 2.5 Results

The ICC analysis indicated acceptable-to-good reliability for all variables: KTK (ICC = 0.660), yo-yo IR1 (ICC = 0.730), Flanker Effect (ICC = 0.984), working memory (ICC = 0.759), inhibition (ICC = 0.726), and cognitive flexibility (ICC = 0.604).

**Figure 4:**
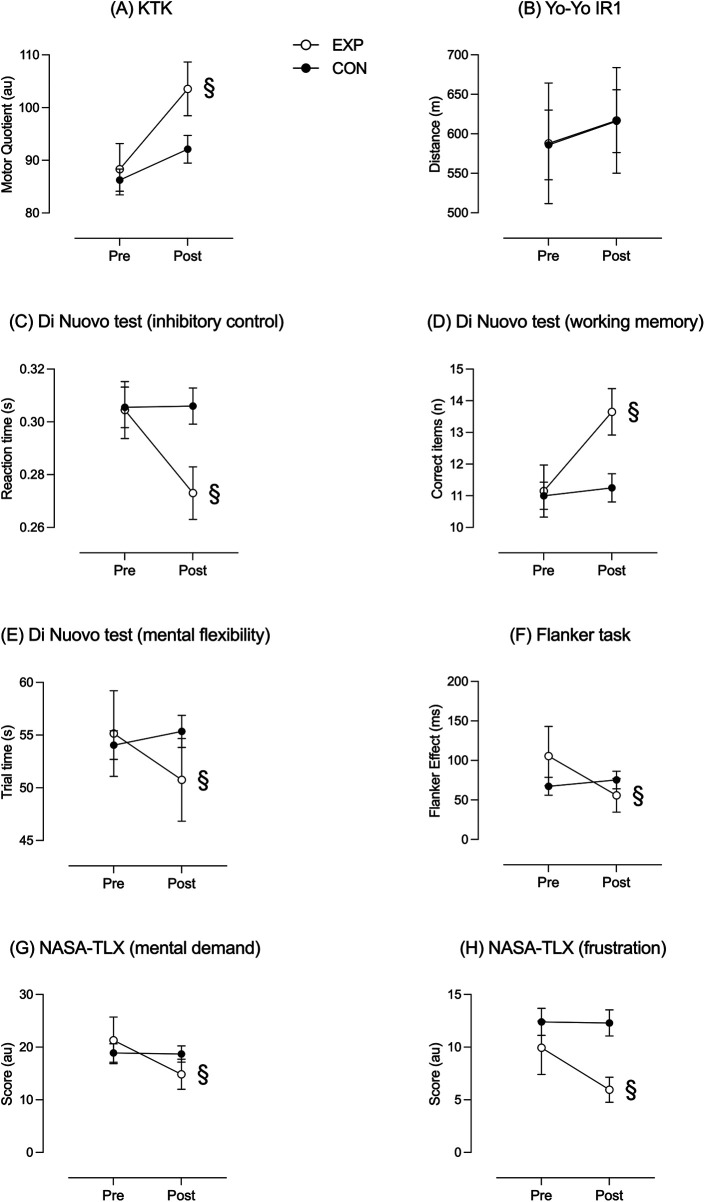
Time × group interactions related to interventions using rugby pedagogy based on ecological and reflective approach (EXP) and traditional methods (CON) on coordination [Panel **(A)**], metabolic conditioning [Panel **(B)**], cognitive aspects [Panels **(C)**, **(D)**, **(E)**, **(F)**], and perceptive aspects [Panels **(G)**, **(H)**]. § = Significant interaction (*p* < 0.05).

Figure 4 displays the most relevant differences between the outcomes from a rugby pedagogy based on ecological and reflective approach (experimental group) and the application of traditional methods (control group).

EXP and CON group did not present significant differences in all the variables at the beginning of the trial (T0). The interactions (time × group) concerning coordination (neuromotor aspects) performed with KTK and expressed as motor quotient (MQ) are shown in Panel A. EXP group significantly increased the coordination (F = 11.53, *p* < 0.002, *η*_p_^2^ = 0.233) at the end of the intervention compared to the CON group. Metabolic conditioning (physiological aspects) is reported in Panel B. EXP and CON groups both significantly increased their performances during time, but there are no significant differences between groups (F = 0.0005, *p* = 0.946, *η*_p_^2^ = 1.214 × 10^−4^). The interactions about executive functions (cognitive aspects) are shown in Panel C: at the end of the intervention, the time expressed in seconds (s) used to perform simple and complex inhibitory control significantly decreased in EXP group (F = 23.80, *p* < 0.001, *η*_p_^2^ = 0.385) compared to the CON group. Panel D shows the working memory ability, expressed as the number of digits remembered, which at the end of the intervention significantly increased in EXP group (F = 22.70, *p* < 0.001, *η*_p_^2^ = 0.374) compared to the CON group. Panel E refers to mental flexibility, expressed in seconds (s), which significantly increased in EXP group (F = 28.645, *p* < 0.001, *η*_p_^2^ = 0.430) as time to perform reduced. Panel F shows interactions about simple inhibitory control performed with the Flanker task: reduced performing times indicate that EXP group improved, differently than CON (F = 4.542, *p* < 0.040, *η*_p_^2^ = 0.107). Finally, Panels G and H report the outcomes from NASA-TLX (perceptive aspects). Significant time × group interactions concerning mental demand can be observed in Panel G (F = 4.287, *p* < 0.045, *η*_p_^2^ = 0.040). Similarly, frustration lowered only in EXP group (F = 2.384, *p* = 0.059, *η*_p_^2^ = 0.131), as displayed in Panel H.

**Figure 5:**
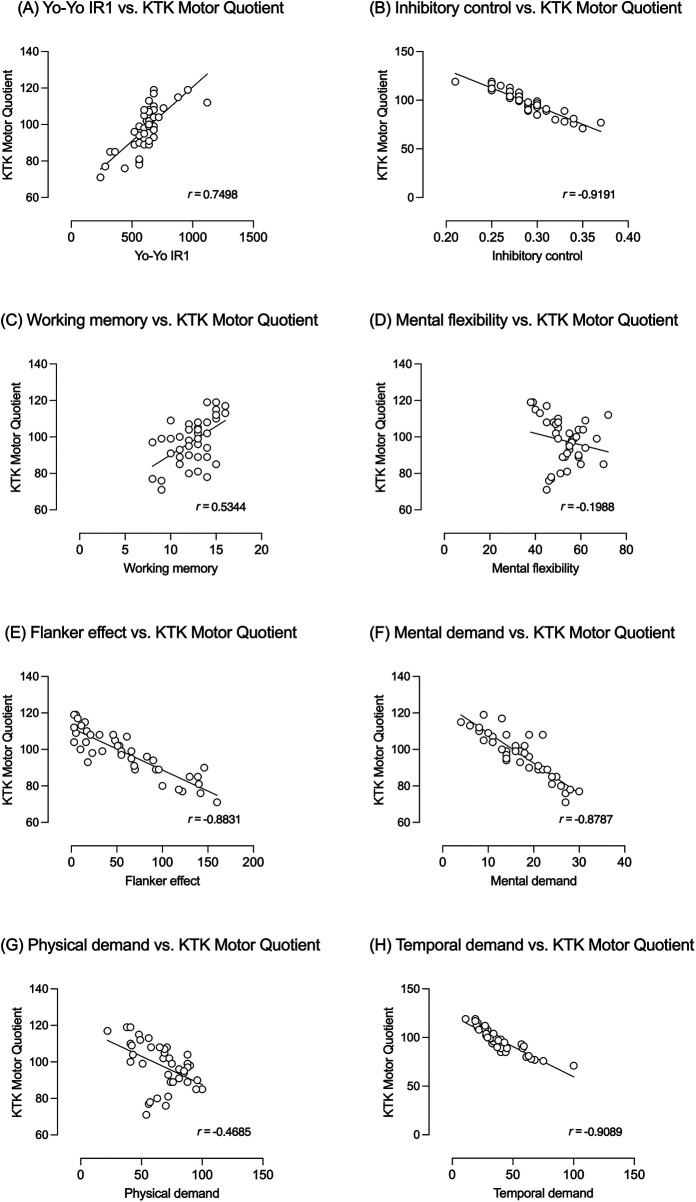
Correlations between neuromotor (coordination—KTK motor quotient) and physiological [metabolic conditioning, panel **(A)**], cognitive [inhibitory control, panel **(B)**; working memory, panel **(C)**; flexibility, panel **(D)**; inhibitory control, panel **(E)**], and perceptive aspects [mental demand, panel **(F)**; physical demand, panel **(G)**; temporal demand, panel **(H)**].

Pearson's correlations analyses (Figure 5) were performed to highlight the red thread between the neuromotor, physiological, cognitive and perceptive aspects. A positive strong correlation (*r* = 0.750; *p* < 0.001) between coordination (neuromotor aspects—KTK motor quotient) and metabolic conditioning (physiological aspects) is shown in Panel A. Correlation with cognitive aspects are reported in Panels B to E. The strong negative correlation with inhibitory control is shown in Panel B (*r* = −0.919, *p* < 0.001). Panel C shows the moderate positive correlation between coordination and working memory (*r* = 0.534, *p* < 0.001), while Panel D the weak negative correlation (*r* = 0.199, *p* = 0.219) with mental flexibility. The strong negative correlation between coordination and inhibitory control measured by Flanker task is shown in Panel E (*r* = −0.848, *p* < 0.001). Finally, correlations between coordination and perceptive aspects are reported in Panels F to H: the negative strong correlation (*r* = −0.879; *p* < 0.001) with mental demand is shown Panel F, those with physical demand (moderate negative correlation; *r* = −0.469; *p* = 0.002) and temporal demand (strong negative correlation; *r* = −0.909, *p* < 0.001), in Panels G and H, respectively.

## 3 Discussion

The primary objective of this research was to investigate the effects of the ecological dynamics approach and active reflection (contemporary learning) on the foundational components that support rugby performance, specifically coordination (neuromotor aspects), metabolic conditioning (physiological aspects), executive function, and workload (cognitive and perceptual aspects). Additionally, the study aimed to explore the interrelationships among these variables to gain a deeper understanding of the integration of the individual dependent variables. The data collected in this study, which compared modern learning approaches—rooted in contemporary paradigms such as ecological dynamics and constructivism—with traditional prescriptive (cognitivist) methods, revealed several significant findings.

In the context of contemporary learning (EXP group), participants exhibited notable improvements in motor control (coordination) and metabolic conditioning (physiological adaptation), alongside enhancements in working memory, mental flexibility, and inhibitory control (cognitive aspects), in line with the studies of (27, 81, 82). These improvements highlight the impact of active engagement and adaptive learning environments, as the EXP group demonstrated better integration of neuromotor, physiological, and cognitive functions. Active reflection and the ecological dynamics approach encourage athletes to engage in reflective thinking about their actions, fostering greater learning and adaptation (9).

Additionally, there was a reduction in perceived effort, including mental demand and frustration, consistent with previous research on representative and learner-centered training environments (27, 83). This reduction is important as it suggests that non-prescriptive, contemporary learning models may reduce mental fatigue and enhance athletes' ability to perform under pressure, making the training process both more effective and sustainable.

The results also revealed correlations between coordination (neuromotor aspects) and metabolic conditioning (physiological aspects), as well as between coordination and cognitive functions, including working memory, mental flexibility, and simple inhibitory control. Further correlations were identified between coordination (neuromotor aspects) and perceived effort, including mental demand, physical demand, and temporal demand (perceptual aspects), supporting integrative models of motor–cognitive coupling under conditions of physical and informational load (43, 84). These findings underscore the importance of understanding the dynamic interaction between physical, cognitive, and perceptual components in performance outcomes. It supports the view that enhancing one area (e.g., coordination) can have ripple effects on other aspects, such as physiological and cognitive functioning, which are critical in complex sports like rugby.

These findings are well-aligned with existing literature that advocates for learning models grounded in ecological dynamics and the constraints-led approach. According to Davids et al. (33) and Chow (9), motor learning arises from the continuous interaction between the individual, the task, and the environment, promoting the simultaneous development of coordination (neuromotor aspects), physiological adaptations, and decision-making processes. This continuous interaction creates a more adaptable and holistic learning process, ensuring that athletes are better prepared to respond to the unpredictability of the sport.

Within this framework, the observed improvements in the EXP group in both motor control and metabolic conditioning suggest that non-prescriptive learning environments foster more integrated and functional adaptations compared to traditional training approaches (36, 85, 86). This conclusion is particularly significant in sports like rugby, where the demands are multifaceted, and performance is influenced by a combination of neuromotor coordination, physiological endurance, and cognitive decision-making. The EXP group's enhanced abilities in all three areas suggest that ecological dynamics can foster more comprehensive skill development, integrating physical, mental, and perceptual capacities.

In the context of rugby, coordination quality is a critical determinant of performance, underpinning key actions such as evasion, tackle execution, ruck entry, passing accuracy under fatigue, and ball protection in contact situations (87). As rugby is a high-intensity sport that demands quick decision-making, accurate movement patterns, and the ability to execute complex skills under pressure, the development of coordination and metabolic conditioning becomes essential for maintaining high levels of performance. The concurrent enhancement of metabolic conditioning observed in the EXP group further supports previous findings that game-based and nonlinear pedagogical approaches can elicit physiological loads comparable to, or even exceeding, those of traditional conditioning drills, while maintaining a high degree of task representativeness (88–90). This finding indicates that contemporary learning environments not only improve motor control but also contribute significantly to the athlete's ability to withstand the physiological stresses of the game.

These results underscore the value of ecological dynamics and contemporary learning frameworks in optimizing performance-related factors in rugby, suggesting that non-prescriptive training models can facilitate more holistic adaptations in both motor control and physiological conditioning. The enhanced coordination and metabolic conditioning observed in the EXP group support the idea that ecological dynamics-based training environments, which replicate game scenarios more closely (representative design), provide superior conditioning benefits compared to traditional, prescriptive methods. This is particularly relevant for rugby, where players are required to perform a wide range of complex movements under varying physical conditions. The enhancement of executive functions—specifically working memory, cognitive flexibility, and inhibitory control—observed in the EXP group is consistent with the findings of Diamond (91) and Best (92), who demonstrated that complex, variable, and cognitively demanding motor activities are particularly effective in promoting executive function development.

This study suggests that contemporary learning paradigms grounded in ecological dynamics and active reflection are highly effective in promoting coordinated neuromotor responses, enhancing physiological conditioning, and fostering cognitive flexibility and executive function in rugby players. The reduction in perceptual workload (lower perceived mental demand and frustration) observed in the EXP group is consistent with the concepts of self-organization and perceptual attunement described by (93) and later applied to sport by Araújo and Davids (94). Learning environments that encourage exploration and reflection-in-action enable athletes to become more sensitive to task-relevant environmental information, thereby reducing non-functional cognitive load and improving perceptual efficiency (9, 32, 95). In addition, the reduction in perceptual workload is also highly relevant to rugby pedagogy. Ecological learning environments facilitate perceptual attunement to key informational variables such as opponent positioning, defensive spacing, and support lines, enabling players to act more efficiently with lower cognitive strain (96–98). This may contribute to improved emotional regulation and resilience during high-pressure game situations, such as defensive sets near the try line or rapid turnovers (99).

The correlations observed between coordination (neuromotor aspects), metabolic conditioning (physiological aspects), and executive functions further support an integrative model of motor and cognitive development, particularly in rugby performance. These findings align with the long-term athletic development framework proposed by Myer, Faigenbaum (44) and Myer, Jayanthi (100), which highlights neuromuscular control as a foundational quality supporting both physical robustness and cognitive efficiency, including the regulation of perceptual workload. Similarly, other studies describe motor coordination as a “bridge” between motor and cognitive development, especially in complex sport contexts (84, 101, 102). In contrast, the results observed in the CON group align with previous research on traditional, prescriptive training approaches. While such methods are effective in enhancing metabolic conditioning (103, 104), they demonstrate limitations in fostering coordination, executive functions, and perceptual workload regulation. Training that emphasizes isolated physical drills and coach-led instruction, while neglecting perception–action coupling and reflection-in-action, appears less capable of promoting integrated development and learning transfer (86, 105, 106). Overall, these findings are consistent with robust scientific evidence derived from systematic reviews and meta-analyses, which attribute to Game-Centred Approaches—particularly Teaching Games for Understanding and the Tactical Games Approach—superior and more integrated effects on the development of motor coordination, physiological conditioning, and perceptual-cognitive abilities compared to traditional approaches (57–60).

Overall, the findings of the present study support a rugby-specific, holistic model of player development, in which neuromotor, physiological, cognitive, and perceptual dimensions are deeply interconnected, consistent with systems thinking and complex adaptive systems theory (107, 108). The ecological-dynamic and reflective pedagogical approach (constructivist approach) emerges as an effective strategy for enhancing game ability, fostering transferable skills, and preparing players to cope with the complex, uncertain, and physically demanding nature of rugby. However, although the connections among various domains are widely recognized, their development does not necessarily occur simultaneously. In fact, an individual may improve in one domain in response to specific training stimuli, without corresponding positive changes in others, particularly when a sufficient level of adaptation has already been achieved.

### 3.1 A conceptual laboratory

To enable further conceptual exploration that complements the findings of the quantitative study, we propose a thought experiment based on an intuitive logical model. The conceptual laboratory (109–111) represents a useful tool for enhancing the understanding of scientific problems through logical and conceptual reasoning. In the present paper, the analysis of the selected variables gives rise to several complex issues, consistent with the study of nonlinear and adaptive systems in sport and education (32, 33). Consequently, the conceptual laboratory aims to: (i) clarify the fundamental concepts underlying the principles of the intervention; (ii) test the internal consistency of a theory based on an integrated approach; (iii) highlight the paradoxes that emerge from the logical consequences of a separatist view of the two approaches; and (iv) stimulate theoretical intuition by integrating it with quantitative empirical data which, if considered in isolation, may be insufficient to capture the complexity of the educational–sporting phenomenon (108).

The thought experiment compares three theoretical conditions: (i) subjects with access to unconscious visual information, as observed in blindsight phenomena (112) (Weis Krantz, 1996; (ii) players trained according to an ecological-dynamic approach, oriented toward the direct perception of affordances (36, 93); and (iii) subjects or systems relying on rigid cognitive patterns and predefined representational strategies (113).

Blindsight phenomena demonstrate that certain motor actions—such as spatial orientation and obstacle avoidance—can be guided by unconscious visual information in the absence of subjective perceptual experience (114, 115). Although limited in precision and in the anticipation of complex situations, these phenomena indicate that action control does not necessarily depend on conscious symbolic representations. Moreover, under stressful conditions characterized by high situational complexity, these subjects tend to lose their spontaneous perceptual abilities, supporting the view that conscious reflection alone is insufficient for adaptive performance (116–118).

In highly complex game contexts, the effectiveness of action depends less on the execution of pre-programmed patterns and more on the ability to continuously adapt to disturbances generated by interactions with other players (33, 94). The direct perception–action coupling typical of athletes trained within an ecological-dynamic framework enables rapid responses, fluid trajectories, and real-time motor reorganization, whereas overly prescriptive strategies tend to constrain adaptive flexibility (32, 119).

To further clarify this point, consider a humanoid hypothetically equipped with complete knowledge of the most effective game strategies. Despite this extensive informational repertoire, such an entity would face significant difficulties when interacting with real teammates and opponents, as the game environment produces a potentially infinite range of situational configurations, characteristic of complex adaptive systems (108, 120). These include creative and emotionally driven situations rooted in the originality, flexibility, and fluency of human players, which cannot be fully codified into standardized rules or plans (86, 121). Within this context, human situational sensitivity—largely unconscious, embodied, embedded, and enacted—enables the perception of emerging affordances and supports flexible responses to contextual demands (36, 122). Consequently, the effectiveness of sporting action cannot be explained solely in terms of explicit mental representations but instead requires a dynamic model of cognition grounded in the continuous and flexible interaction between organism and environment (86, 121). This perspective does not deny the role of cognition or representational processes. Representations play a crucial function during less time-constrained phases, such as strategic planning, learning, and post-action reflection (123, 124), by providing shared conceptual frameworks that facilitate collective coordination. During real-time action, however, behavior is predominantly regulated by direct perception and unconscious control processes (14, 119, 125). Effective performance thus emerges from the functional integration of representational cognition and the ecological dynamics of perception.

The theoretical implications suggest that self-awareness in play primarily emerges as a pre-reflective, embodied, and relational phenomenon, rather than as the outcome of symbolic and rational cognitive processing (126–128). Furthermore, the integration of spontaneous adaptation with reflective processes (typical of constructivist approach) is proposed as an effective training model for recognizing and internalizing the languages and principles of the game (40, 86, 129).

**Figure 6:**
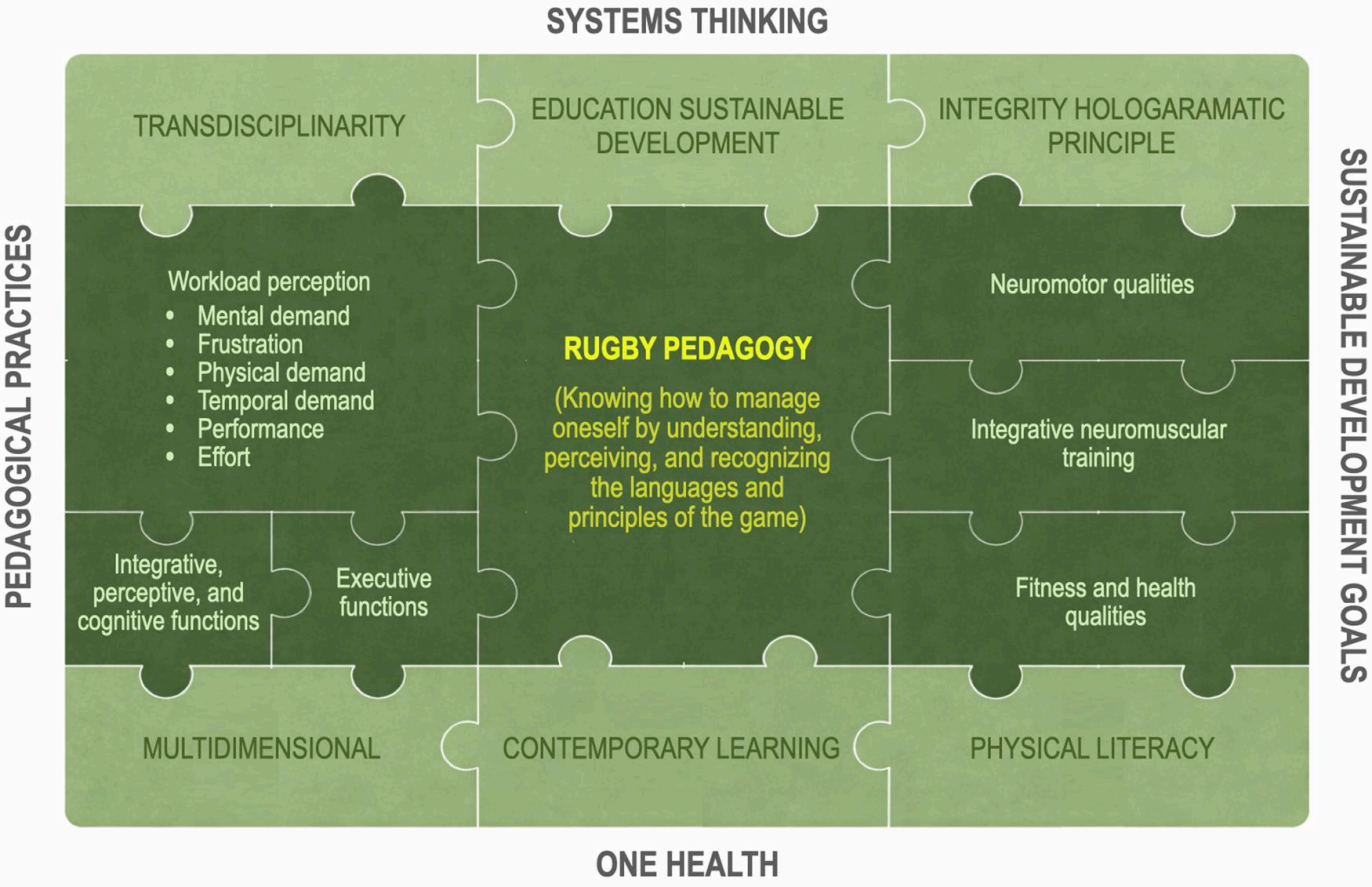
Representation of the rugby pedagogy framework. The puzzle represents a complex system in which several macrosystems with their subsystems interact. Every piece of the puzzle constitutes a peculiar element that interacts with the other parts (pieces) of the complex system (puzzle).

Figure 6 illustrates the concept of rugby pedagogy (the central element of the figure), which is grounded in self-regulation within an integrative embodied–mind framework (14, 122). This framework is based on the functional integration of neuromuscular, perceptual, and cognitive processes (32, 33) and provides the theoretical foundation for understanding the language, logic, and principles of the game, thereby supporting the development of individual awareness (126). From this foundational level, learners progressively construct a broader systemic awareness that extends beyond the individual to include interaction with the surrounding environment, consistent with enactive and ecological accounts of cognition (36, 130). This perspective incorporates the one health approach (131), pedagogical practices oriented toward the sustainable development goals (132), and a conception of sport as a complex adaptive system characterized by dynamic and interdependent relationships among multiple components, in line with systems thinking (1, 108). At a second internal level of this complex system, depicted in light green, contemporary learning (ecological dynamics and constructivist approach) paradigms underpin sustainable rugby pedagogy. These include physical literacy as a determinant of lifelong engagement in health-enhancing physical activity (53, 121, 133); multidimensionality as an educational approach that prioritizes holistic development over exclusively performance-based outcomes (43); transdisciplinarity, which conceptualizes the body and movement as primary sources of cognition and consciousness (122, 134); the holographic principle, emphasizing that interventions at any single level of the system generate effects across the entire structure (135); and education for sustainable development, oriented toward learning both for and through embodied experience (42).

This exploratory contribution proposes an integrated theoretical framework linking neuroscience, phenomenology, and education to demonstrate how the development of self-awareness—understood as an embodied and self-regulated experience—constitutes the neurocognitive and phenomenological foundation of ethical, ecological, and global consciousness (126, 128, 136, 137). Neuroscientific evidence suggests that consciousness emerges from the interaction of bodily, attentional, and executive systems, involving sensorimotor, interoceptive, and emotional networks (138–141). Phenomenology further clarifies how the sense of self is rooted in prereflective embodied awareness and how subjectivity is intrinsically relational (126, 128, 136). On this basis, the paper argues that mature self-awareness fosters empathy, recognition of interdependence, and systems thinking, thereby reducing the dichotomy between the individual and the environment (1, 142). These capacities make complex paradigms such as *One Health* agenda and the sustainable development goals cognitively and emotionally accessible, as they require long-term vision, self-regulation, and collective responsibility (42, 131).

Within the educational domain, this exploratory framework suggests that practices grounded in mindful movement, attention, and experiential learning can facilitate the transition from individual self-formation to a form of global consciousness oriented toward sustainability (86, 143). This model requires systems thinking, long-term orientation, self-regulation, and collective responsibility—capacities that are not merely technical but emerge from a stable and integrated form of embodied consciousness (33, 122). In this sense, educating embodied awareness through contemporary rugby pedagogy is not an ancillary objective but a structural prerequisite for addressing contemporary ethical, health-related, and environmental challenges (40).

### 3.2 Limitations

Some limitations in the present study should be highlighted. First, given that the central aim of the study was to verify whether a reflective dynamic ecological approach fostered the integrated development of the mental, physical, coordinative, and perceptual correlates of young players, it should be noted that the instructor's own characteristics may have influenced the results. However, this is a distinctive feature of the human sciences, which cannot be entirely overcome in educational and training contexts. Second, although the number of participants was appropriate to ensure adequate statistical power, the inclusion of a single rugby club may limit the generalizability of the results to similar sporting contexts. Third, the correlation analysis aimed to assess the associations between physiological, cognitive, and motor domains. Using this approach, our intention was not to demonstrate causality but rather to provide an initial exploratory insight into the relationships among various domains. Finally, we acknowledge that assessing physiological, cognitive, and motor aspects separately may appear inconsistent with an integrated theoretical perspective on which the present study was based and may represent a limitation; therefore, future methodological approaches capable of capturing a true integration of all domains, potentially within a single test, are needed.

## 4 Conclusions

A learning approach based on contemporary paradigms applied to an intervention of rugby pedagogy, is effective to improve young athletes' motor control (coordination aspects), metabolic conditioning (physiological aspects), as well working memory, mental flexibility, and inhibitory control (cognitive aspects), reducing perceptive effort, mental demand and frustration. This ecological and reflective approach favours the development and the integration of the different capacities (mental, coordinative, perceived, and physical) better than a traditional prescriptive approach.

Within the new conceptual laboratory proposed in this study to better address the complexity of the educational–sporting phenomenon, the fundamental principles of rugby—advancing, supporting, and continuity—can be interpreted as embodied structures of meaning capable of fostering the development of a global and holistic awareness. Such awareness is valuable not only for sporting performance but also for sustainable adaptation to the complex environments of everyday life (144, 145), in alignment with the one health principles, systems thinking, and sustainable development goals.

## Data Availability

The raw data supporting the conclusions of this article will be made available by the authors, without undue reservation.
