# Supplementary material for: Systemic relationships between ecological-dynamic approach and active reflection: psycho-neuro-motor, cognitive, and physiological interactions in rugby pedagogy
Source: Front Sports Act Living. 2026 May 14;8:1804050. doi: 10.3389/fspor.2026.1804050 (PMC13216005; doi:10.3389/fspor.2026.1804050)
Supplement: Supplementary file 1 [file Table1.docx]

**Appendix 1. Synthesis of concepts and literature main findings of the main variables considered in the study**

| **Variable** | **Topic** | **Main Findings/Insights** |
| --- | --- | --- |
| Neuro-motor aspects | Improvement in neuro-motor control enhancing physiological condition | Coordination and perceptual skills, classified as neuromotor skills, are essential for cardiovascular, metabolic health, and functional performance (Garber et al., 2011; Lubans et al., 2016; Pesce et al., 2016). These skills develop in a non-linear manner influenced by age and environmental factors (Thieschäfer & Büsch, 2022). |
| Physiological aspects | Improvement in physiological condition enhancing neuro-motor control | High levels of physical conditioning improve coordination and neuromotor control, with exercise enhancing cerebral perfusion and brain plasticity (Bavelier & Neville, 2002; Myer et al., 2011; Lubans et al., 2016; Pesce et al., 2016). |
| Perceptual and Cognitive aspects | Improvement in self-perception enhancing adaptive interactions with other and the environment | Sports pedagogy promotes psychophysical literacy through embodied experiences and movement, enhancing self-awareness, executive functions, and the ability to perceive different type of psyco-neuro-motor and social adaptive effort (Diamond, 2013; Rudd et al., 2021; Damiani et al., 2019; Chakraborty et al, 2023; Renshaw et al., 2010; Klotzbier & Schott, 2025). |
| Integration | Interactive improvement of psycho-neuro-motor, cognitive and physiological condition within game-centred approach | An ecological and reflective approach to game-centred pedagogy can improve neuromotor, physiological, cognitive, and perceptual aspects, promoting coordination, fitness, executive functions, and reducing fatigue perception in youth (Gonzales, 2024; Breed et al., 2025; Ortiz et al., 2023; Wang et al., 2024; Capra and Luisi, 2014, McLean et al., 2025). |
