# Supplementary material for: Systemic relationships between ecological-dynamic approach and active reflection: psycho-neuro-motor, cognitive, and physiological interactions in rugby pedagogy
Source: Front Sports Act Living. 2026 May 14;8:1804050. doi: 10.3389/fspor.2026.1804050 (PMC13216005; doi:10.3389/fspor.2026.1804050)
Supplement: Supplementary file 2 [file Table2.docx]

**Appendix 2. Example of intervention contents**

| **Group** | **Activity** |
| --- | --- |
| Experimental (Ecological dynamics approach) | - Individual and collective technical exercise with the use of a mask aimed at covering the dominant eye to go to work on reprogramming visuo-spatial perception (variations on the organism). The expert gave instructions that players try to follow. - Situational association small-sided games are implemented using different colors to indicate target try zones (yellow, red, blue, white) within a 6 × 6 m square. Players compete in a 1v1 format, starting from the center of the square, while the instructor designates the target try zone (yellow, blue, red, or white) that must be reached. A variation of this activity involves word–color association: yellow–sun, red–fire, blue–water, and white–snow. In this case, the instructor calls out the word rather than the color, requiring players to cognitively associate the stimulus with the corresponding target. Similar exercises can be adapted to larger formats, such as a 2v1 setup in an 8 × 10 m area or a 4v2 setup in a 15 × 20 m area. In these configurations, try lines are positioned along the shorter sides of the field, while players begin from the center of the longer side. The instructor may further increase task complexity by indicating numerical cues (e.g., even numbers correspond to the red try zone, odd numbers to the white try zone), potentially incorporating simple mathematical operations to determine the target. The players adapted their decision to the command and the environment. - Small-sided or formal game with the use of several balls at the same time, with different shapes and sizes, essential for developing individual technique (e.g., passing with hands with the rugby ball, passing with feet with the round ball) (variation on tasks). The expert mediated the discovery of new coordination and strategies, motivating them with innovative proposals. Players reflected after practice, sharing new discoveries and strategies. - Formal and adapted games using a game-centered approach where expert formulated questions designed to stimulate play intelligence and comprehension (cognitive workload). |
| Control (traditional approach) | - Individual and collective technical exercises oriented to the development of rugby skills. The instructor directed the practice, and players followed the instructions. - Situational small-sided games in a 1vs1 in a 6x6m square field (the player with the ball tried to score without being tackled), in a 2vs1 in a 8x10m rectangular field starting from the long side or 4vs2 in a 15x20m rectangular field starting from the center of the long side, aimed at stimulating tactics and repetitions of the general game situation at reduced ranks. The intructor guided the exercise, determining the executive rhythm (starting the games). The players adapted their decision to the situation. - Formal games starting with specific game situations (e.g., penalty, touch, drop-out, scrum). The instructor provided tactical information about the game situation, and players tried to play from different game phases. - Formal situational game where the instructor acted as referee and players played rugby with all the rules. The instructor directed the game and provided information on tactical and technical mistakes. Instructors determined the game rhythm, accelerating or decelerating it to regulate the physical intensity. |
